# Magnesium Modifies the Structural Features of Enzymatically Mineralized Collagen Gels Affecting the Retraction Capabilities of Human Dermal Fibroblasts Embedded within This 3D System

**DOI:** 10.3390/ma9060477

**Published:** 2016-06-15

**Authors:** Federica Boraldi, Angelica Bartolomeo, Giulia Annovi, Romain Debret, Daniela Quaglino

**Affiliations:** 1 Department of Life Sciences, University of Modena and Reggio Emilia, Via Campi 287, Modena 41125, Italy; federica.boraldi@unimore.it (F.B.); angelica.bartolomeo@unimore.it (A.B.); annovigiulia@gmail.com (G.A.); 2 Laboratory of Tissue Biology and Therapeutic Engineering (LBTI), UMR5305 CNRS/UCBL, Lyon 69366, France; romain.debret@ibcp.fr

**Keywords:** collagen type I, alkaline phosphatase, fibroblast, three dimensional gel, morphology, mineralization

## Abstract

Mineralized collagen gels have been developed as *in vitro* models to better understand the mechanisms regulating the calcification process and the behavior of a variety of cell types. The vast majority of data are related to stem cells and to osteoblast-like cells, whereas little information is available for dermal fibroblasts, although these cells have been associated with ectopic calcification and consequently to a number of pathological conditions. Therefore, we developed and characterized an enzymatically mineralized collagen gel in which fibroblasts were encapsulated within the 3D structure. MgCl_2_ was also added during gel polymerization, given its role as (i) modulator of ectopic calcification; (ii) component of biomaterials used for bone replacement; and (iii) constituent of pathological mineral deposits. Results demonstrate that, in a short time, an enzymatically mineralized collagen gel can be prepared in which mineral deposits and viable cells are homogeneously distributed. MgCl_2_ is present in mineral deposits and significantly affects collagen fibril assembly and organization. Consequently, cell shape and the ability of fibroblasts to retract collagen gels were modified. The development of three-dimensional (3D) mineralized collagen matrices with both different structural features and mineral composition together with the use of fibroblasts, as a prototype of soft connective tissue mesenchymal cells, may pave new ways for the study of ectopic calcification.

## 1. Introduction

Formation of hard connective tissue, such as dentin, bone and cementum, involves calcium phosphate deposition within a collagen matrix. Apparently, similar mechanisms may take place also in the aberrant calcification of soft connective tissues; nevertheless, regulatory mechanisms, especially in pathologic conditions, are still elusive.

Therefore, exploiting strategies capable of regulating mineral deposition demands a better understanding of cell behavior in a calcified context and requires the development of simple and reproducible models. Due to the complexity of *in vivo* biomineralization, different *in vitro* models are frequently applied, most of them providing pro-mineralizing factors’ supplementation into the culture medium.

Despite the use of a variety of natural macromolecules and/or synthetic polymers as organic matrices for hydroxyapatite mineralization, collagen type I is the most recurrent embedding substrate for cell encapsulation due to its biocompatibility and similarity to native extracellular matrix [1,2]. Moreover, mineralized collagen gels, mimicking bone composition, have been extensively studied for bone tissue engineering applications [3,4]. It is well known that collagen *per se* does not induce mineral formation, requiring the presence of ions/salts [5], of non-collagenous proteins [6] and/or of amorphous calcium phosphate [7]. Nevertheless, an appropriate collagen structure is necessary to guide crystal growth and organization [5], allowing proper collagen-mineral interactions, infiltration of calcium and phosphate within collagen fibrils, as well as hydroxyapatite nucleation [8,9,10]. Collagen gel mineralization has been either obtained by incorporating hydroxyapatite [11,12], by seeding cells cultured for several days in the presence of pro-osteogenic supplements as β-glycerophosphate and ascorbic acid [13,14,15], or by preparing collagen/calcium phosphate multilayers, where mineralization is the result of enzymatic reactions [16] and cells are only spread on the matrix surface. Interestingly, stem cells (*i.e.*, dental pulp stem cells, bone marrow mesenchymal stem cells or adipose stem cells) as well as osteoblasts or smooth muscle cells have been widely investigated, whereas other cell types (*i.e.*, fibroblasts) have captured only little attention, even though they regulate connective tissue biosynthesis and organization and are associated with ectopic calcification, and consequently to a large number of pathologic conditions [17].

The aim of this study was to produce and characterize enzymatically mineralized collagen gels in which cells (human dermal fibroblasts–HDF) were embedded within the three-dimensional (3D) structure.

**Figure 1:**
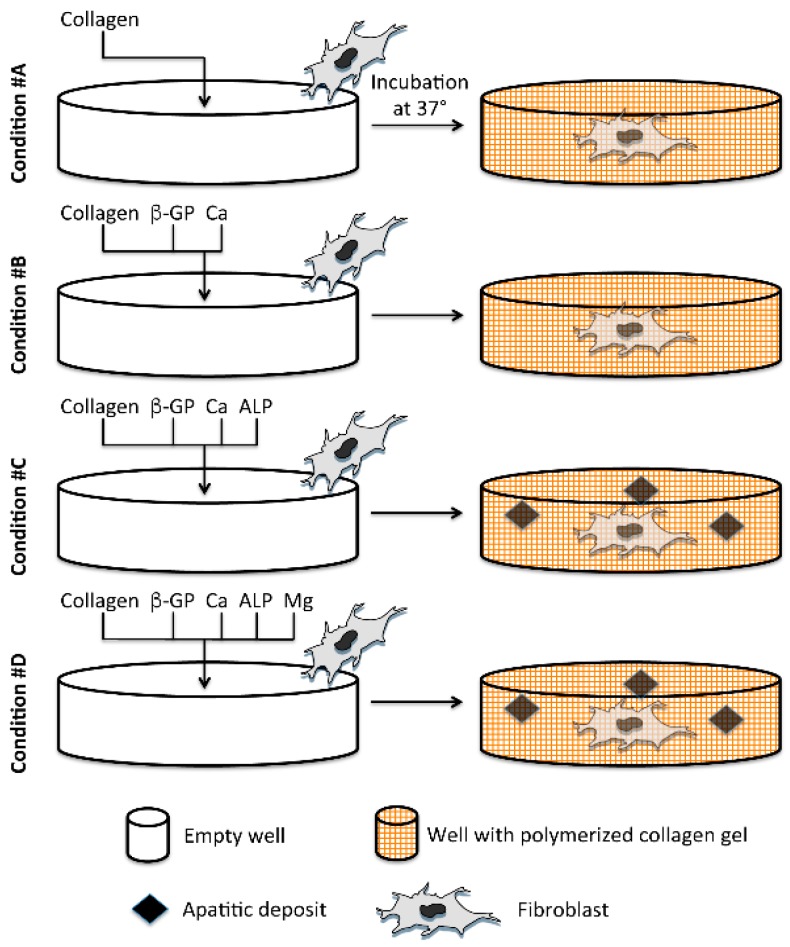
Experimental protocol for collagen gel preparation. Condition **#A** is represented by a standard collagen gel and, in sequence, the following components are added: β-glycerophosphate (β-GP) and CaCl_2_ (Ca) (condition **#B**), Alkaline Phosphatase (ALP) (condition **#C**) and MgCl_2_ (Mg) (condition **#D**). Presence of ALP in #C and #D promotes the cleavage of β-GP to release phosphate that can react with calcium ions to form apatitic deposits. In all experimental conditions, fibroblasts are added before collagen gel polymerization.

To achieve this goal, calcium salts (CaCl_2_) and β-glycerophosphate (β-GP), as a source of phosphate, were mixed into the collagen solution in the presence of alkaline phosphatase (ALP), the enzyme that promotes phosphate (P) cleavage from organic phosphate-containing substrates, thus releasing P capable of reacting with calcium ions to form apatitic deposits. Human dermal fibroblasts were added and mixed with the collagen solution before polymerization (Figure 1). Since collagen polymerization is sensitive to a number of variables such as collagen concentration, pH, temperature or presence of other matrix components (*i.e.*, glycosaminoglycans) [18,19,20,21], all experimental conditions were accurately controlled. In the present study, MgCl_2_ was also added to the collagen solution (Figure 1) since it has been suggested that magnesium: (i) interferes with mineral deposition possibly preventing ectopic calcification [22]; (ii) can be a modifier of collagen assembly [23]; and (iii) is present in calcified nodules in clinical contexts [24,25].

## 2. Results

### 2.1. Light and Scanning Electron Microscopy Reveal a Different Structure of Collagen Gels

**Figure 2:**
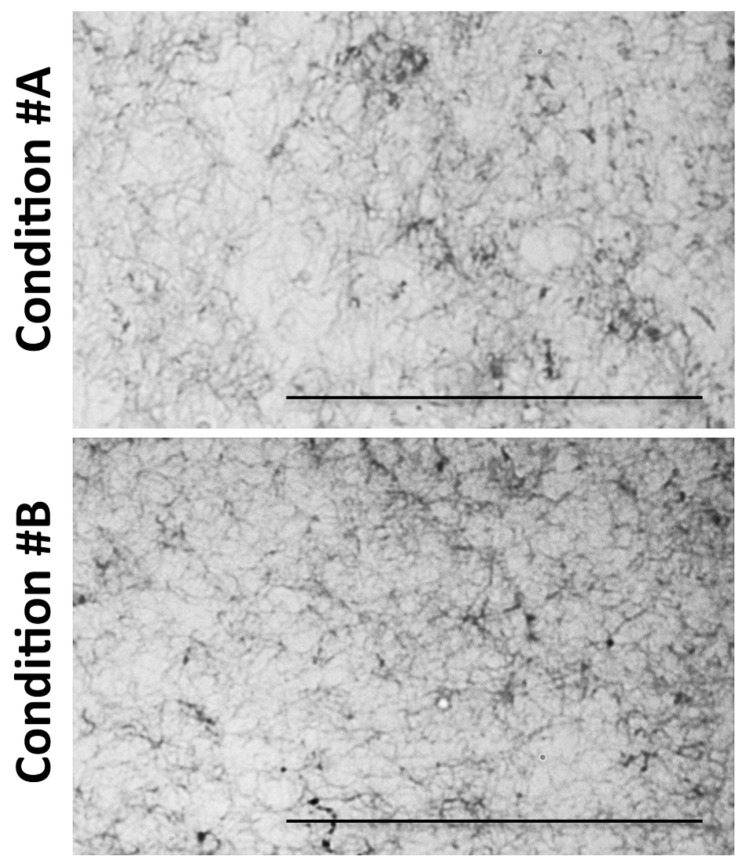

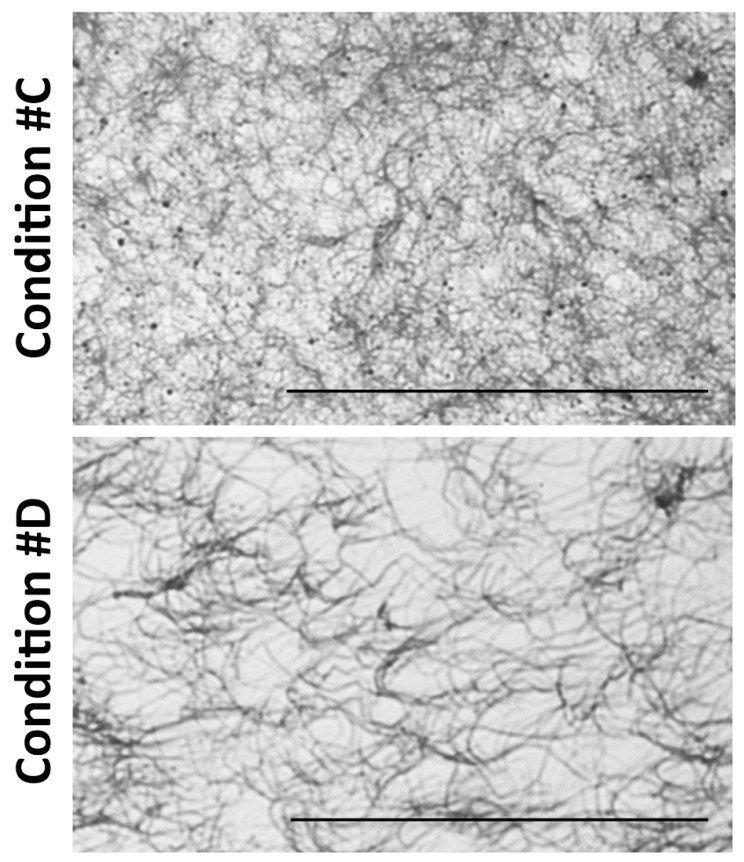
Light microscopy of collagen gels. The fibrillar organization of collagen gels is visualized by light microscopy after Anilin blue staining. Images correspond to the four experimental conditions as in Figure 1. Bar: 100 μm.

Sections of paraffin embedded collagen gels were stained with Anilin blue and observed by light microscopy (Figure 2).

In particular, in conditions #A and #B, collagen fibrils were very similar in size and organization, forming a loose 3D structure. In condition #C, fibrils appeared to be organized in a more compact and intricate network, whereas a completely different 3D organization was noted in condition #D, where MgCl_2_ was added. Magnesium chloride was added at concentrations below 100 mM because higher amounts enhance collagen solubility [26]. Surprisingly, fibrils were thicker in diameter, providing a porous structure with larger empty areas between fibrils.

**Figure 3:**
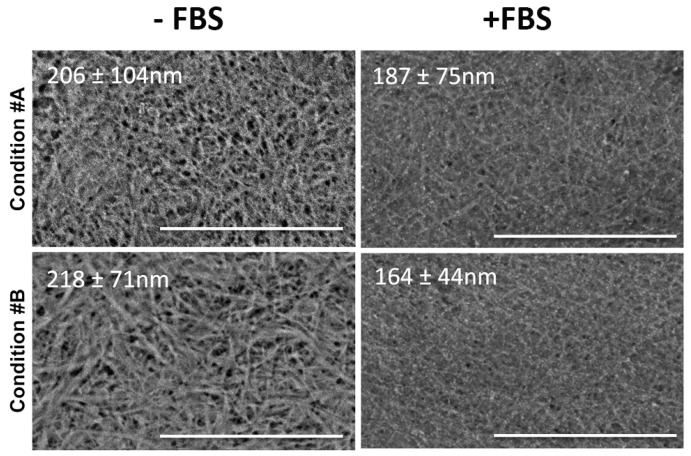

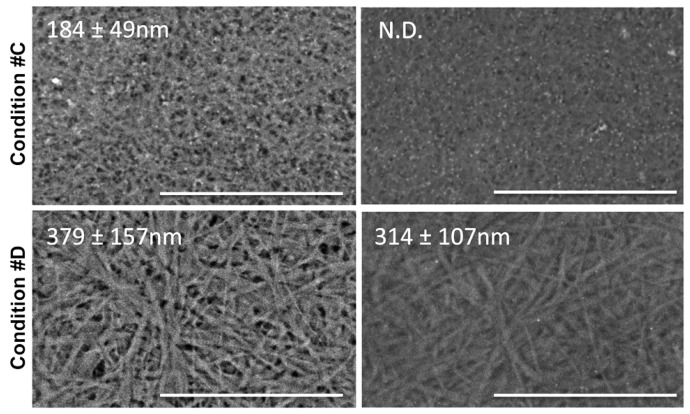
Scanning electron microscopy of collagen gels**.** Collagen gel organization, in the absence/presence of foetal bovin serum (FBS), is investigated by scanning electron microscopy (SEM). Images correspond to the four experimental conditions as in Figure 1. Values represent the mean diameter of collagen fibrils ± SD. N.D. not determined. Bar: 10 μm.

To better visualize the characteristics of collagen gels, samples in the four different experimental conditions were also observed by Scanning Electron Microscopy **(**SEM) (Figure 3).

Moreover, since collagen gels represent suitable 3D substrates to be populated by cells that require serum factors’ supplementation for their survival and adhesion, morphological observations were performed after placing the gels in standard medium (DMEM) in the absence/presence of FBS (Figure 3).

In the absence of FBS, the structural organization of collagen gels and fibrils’ diameters were very similar in conditions #A and #B (206 ± 104 and 218 ± 71 nm, respectively). For condition #C, the mean diameter was not significantly different from previous conditions (184 ± 49 nm), but fibrils appeared more densely packed. In contrast, in condition #D, there were striking differences in the morphology of the fibrils whose diameter was significantly larger (379 ± 157 nm; *p* < 0.001 condition #D *vs.* other conditions) (Figure 3, panel left).

Interestingly, the presence of FBS, added to the medium after gel polymerization, caused an apparently denser matrix, possibly due to serum proteins interacting with collagen fibrils and remaining embedded within the 3D structure; however, diameters of collagen fibrils were not significantly different from those measured in the absence of FBS. It should be noted that, in condition #C, the presence of FBS rendered gels as murky networks, preventing clear discrimination of collagen fibrils (Figure 3, panel right). The addition of Mg significantly increased the mean diameter of collagen fibrils (314 ± 107 nm, *p* < 0.001 condition #D *vs.* other conditions).

Observations by scanning transmission electron microscopy revealed that MgCl_2_ supplementation favored the aggregation of collagen fibrils that appeared adjacent one to the other or laterally fused (Figure S1).

### 2.2. Mineral Deposition in Collagen Gels Requires ALP Activity

**Figure 4:**
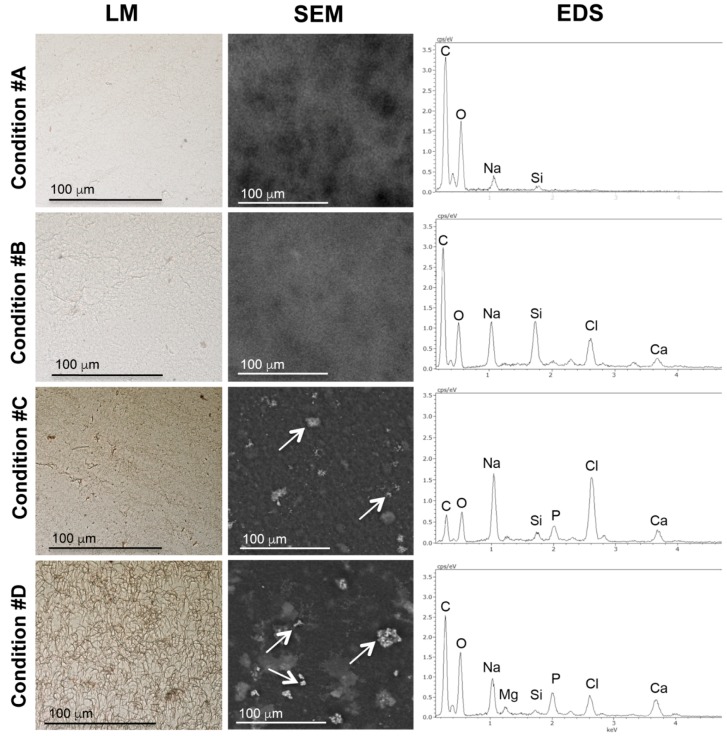
Structural characterization of mineral deposits after 24 h after gel polymerization. The presence of mineral deposits is visualized by Light Microscopy (LM) after von Kossa staining (**brown**). Shape and composition of mineral deposits is shown by SEM and Energy-Dispersive Spectroscopy (EDS) spectra. White arrows indicate apatitic deposits.

Mineral deposition was evaluated on collagen gel sections stained with the von Kossa method and observed by light microscopy (Figure 4).

Twenty-four hours after gel polymerization, paraffin sections from gels prepared in conditions #A and #B were devoid of the typical brown staining associated with phosphate deposits, whereas collagen gels containing exogenously added ALP (conditions #C and #D) were both positive, confirming that P was actually cleaved by ALP from P-containing substrates (*i.e.*, β-glycerophosphate) becoming available to form complexes with calcium.

To confirm the presence of mineral deposits into collagen gels, samples were analyzed by SEM associated with a detector for microanalysis. Distribution of mineral deposits and the corresponding EDS spectra are shown in Figure 4. Gels in conditions #A and #B were devoid of mineral deposits. In condition #A, EDS spectra highlighted the presence of organic matrix components (C and O) and of Na derived from the preparing solution, whereas in condition #B, we also found Ca and Cl, consistently with the addition of CaCl_2_. In both samples the peak of P was absent. In conditions #C and #D, globular deposits were present within the gel, their distribution being rather homogeneous. In addition to the elements already described, EDS analysis revealed that the peak of P, as a result of ALP activity, was specifically detected on mineral deposits. It should be noted that in condition #D, the presence of Mg was prevalent in the globular structures. Although this approach did not allow for defining the characteristics related to the apatitic nature of mineral deposits, data indicate that Mg was present in these structures. Finally, since glass slides were used to immobilize collagen gel during analyses, all samples showed a peak corresponding to Si.

### 2.3. Homogeneous Distribution but Different Morphology of HDF within Collagen Gels

**Figure 5:**
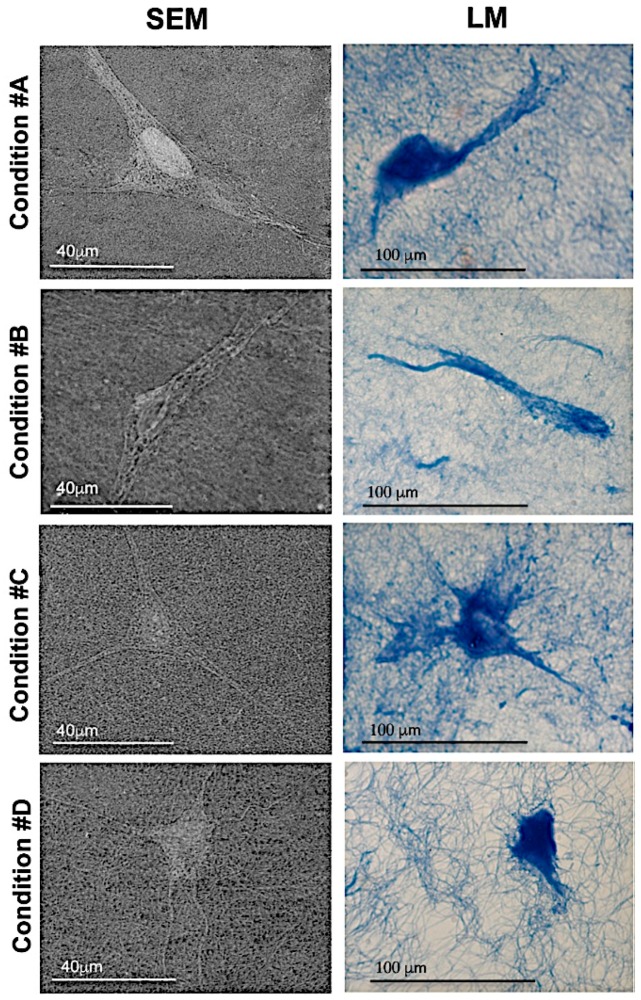
Morphology of fibroblasts embedded within collagen gels. Experimental conditions are those illustrated in Figure 1. Cells are observed by SEM and LM. In condition **#D** cells have a less elongated shape.

The morphology and the distribution of HDF on the surface of collagen gels were assessed by SEM on fixed collagen gels. In all conditions, cells appeared homogeneously distributed; however, in conditions #A, #B and #C, HDF exhibited an elongated shape with extended cytoplasmic protrusions, whereas in condition #D, the cellular body was more globular with thin branches of various lengths (Figure 5). These results were confirmed by light microscopy on cross-sections of collagen gels embedded in paraffin.

### 2.4. Enzymatically Mineralized Collagen Gel Is Cytocompatible

**Figure 6:**
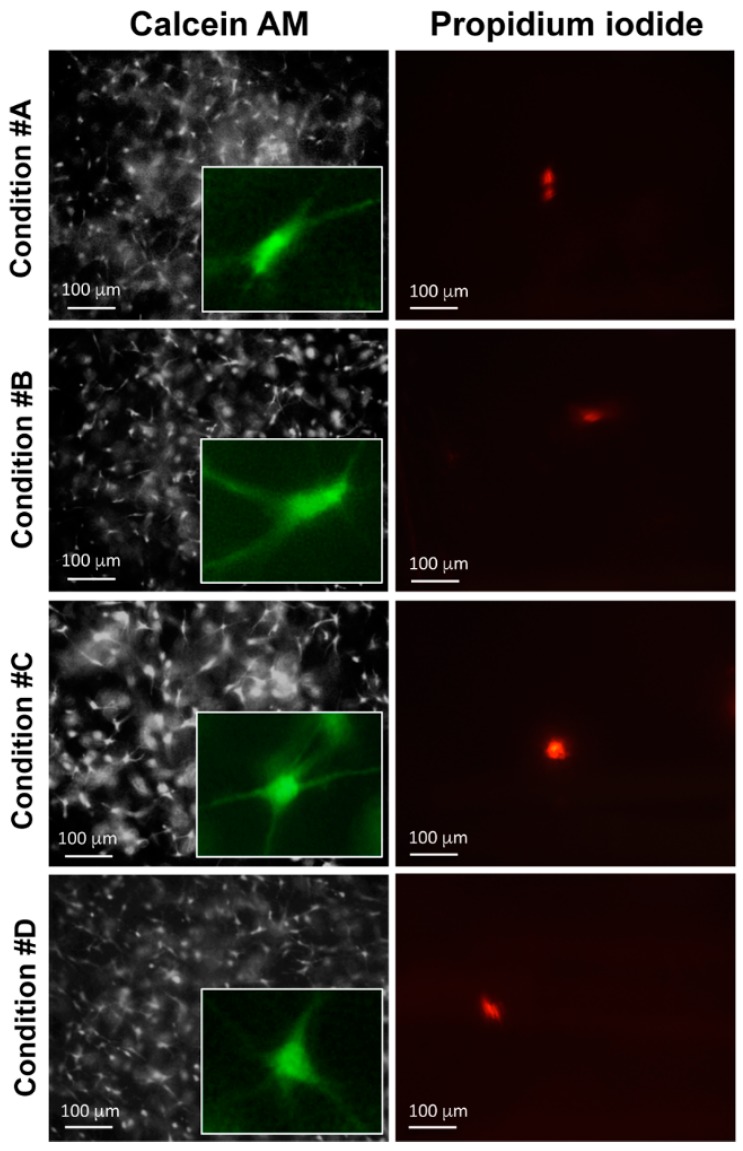
Live/dead fluorescence microscopy of fibroblasts embedded within collagen gels. Experimental conditions are those illustrated in Figure 1. Cell viability is assessed by calcein-AM staining and fluorescence is visible in all conditions. By contrast, only rare dead cells, stained with propidium iodide, are occasionally seen in all samples.

**Figure 7:**
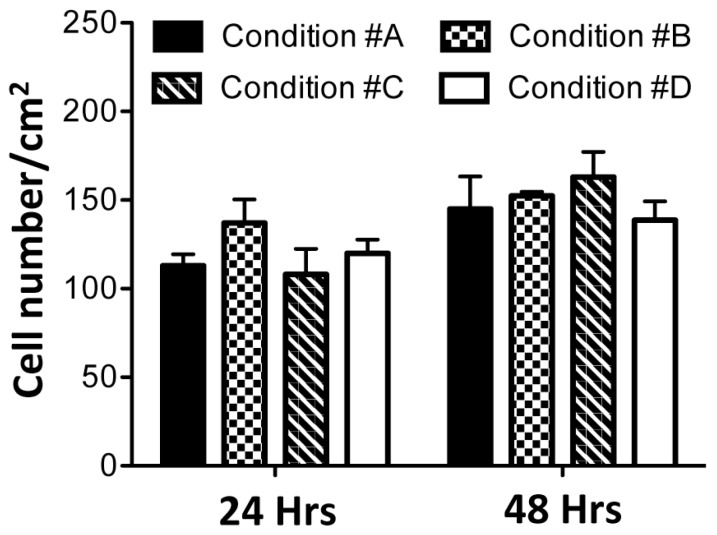
At 24 and 48 h after gel polymerization, viable cells were counted by ImageJ software. Experimental conditions are those illustrated in Figure 1. Values represent the mean of viable cell number/surface area (cm^2^) ± SD.

Calcein-acetoxymethyl (AM) provides a simple, rapid and accurate method to measure cell viability [16]. Live/dead fluorescence microscopy of HDF embedded within collagen gels is shown in Figure 6. Viable cells, stained green, are visible in all conditions after 48 h in culture. As a general observation, there is not a great difference between gels with or without ALP, in the presence or absence of mineral deposits and in the presence or absence of magnesium. Only a few dead cells (1 ± 1/cm^2^), stained by propidium iodide [27], can be seen in all samples (Figure 6). After 24 and 48 h of gel polymerization, the number of viable cells was quantified by Image J software (open source Java image processing program) (Figure 7). No significant differences were noted over time and among the different experimental conditions. Therefore, the presence of different compounds utilized to develop collagen gels, and the presence of apatitic deposits, do not have cytotoxic effects.

Because collagen gels are characterized by a different structure and by the absence/presence of mineral deposits, the ability of HDF to retract the gels has been evaluated.

**Figure 8:**
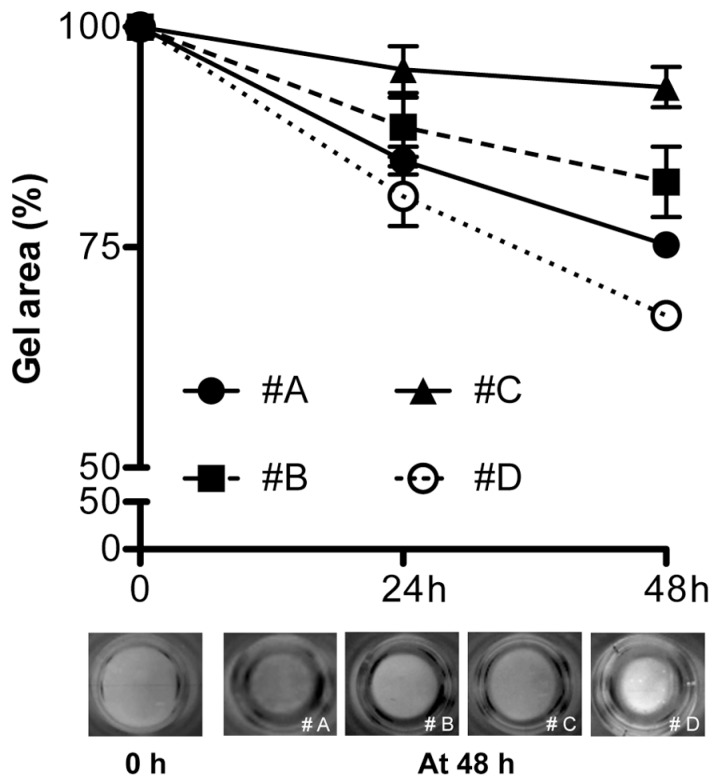
Collagen gel retraction after 24 and 48 h from cell seeding. Experimental conditions are those illustrated in Figure 1. Values represent the mean area of collagen gels ± SEM. Representative images of collagen gels before gel retraction (0 h) and at 48 h (four experimental conditions) are also shown.

As expected, in the absence of serum factors, HDF were not able to contract the gel in any condition, further confirming that serum components are necessary for retraction. In the presence of serum, after 3 h and 6 h in culture, collagen gel diameters did not change significantly compared to the initial phase of the experiments. At 24 h and 48 h in culture, reduction of gel diameters varied depending on the experimental condition. In particular, at 48 h, collagen gels without mineral deposits (#A; #B) were retracted approximately 25% of their original diameter. A reduction of 6% to 35% of the original gel size was observed for conditions #C and #D, respectively (*p* < 0.05 condition #A or #B *vs.* condition #C and #D; *p* < 0.01 condition #D *vs.* condition #C) (Figure 8).

As an additional control, we prepared collagen gels in which mineralization was obtained by salt precipitation after addition of CaCl_2_ and Na_4_P_2_O_7_ (3 mM) at the same concentration used in *in vitro* smooth muscle cell models. Heavy mineralization was rapidly obtained; however, fibroblast viability appeared significantly reduced and gel retraction was negligible (Figure S2).

## 3. Discussion

To better understand the mechanisms regulating the calcification process, either in physiological and/or in pathological conditions, a number of *in vitro* culture systems have been already described, including a few in which mineralization is obtained by addition of alkaline phosphatase (ALP) during gel preparation [11,12].

The novelty of the present study is that collagen gels were prepared in the absence of crosslinking agents and cells were not prepared separately from the mineralized collagen gel, but added prior to polymerization, thus causing their embedding within the 3D structure. Moreover, by adding different components to the collagen solution, we have obtained gels with various structural characteristics.

To our knowledge, the most frequently used cell lines are represented by osteoblast-like cells [28] and dental pulp stem cells [29], *i.e.*, cells that are predisposed to produce and to be in contact with a calcified extracellular matrix, or by vascular smooth muscle cells that are known to shift towards an osteoblast-like phenotype [30]. Dermal fibroblasts play a key role in soft connective tissue homeostasis, can be involved in ectopic calcification and, if cultured in two-dimensional (2D) systems for at least three weeks in the presence of pro-mineralizing factors, can produce a calcified matrix [31,32]. Nevertheless, no data are available on these cells when embedded within a calcified matrix.

In the present study, calcification, as demonstrated morphologically and by microanalysis, was enzymatically induced by exogenously added ALP in a shorter time (within 24 h) [12] compared to other *in vitro* models (2–3 weeks), where mineralization occurred after prolonged immersion of crosslinked polymerized gels in a Ca plus β-glycerophosphate solution [33,34]. Moreover, results demonstrate that fibroblasts, although in a non-physiologic context, can adapt themselves to the mineralized environment.

In addition, collagen gel structure reflects the experimental condition and, in particular, the presence of magnesium. Besides variation in collagen concentration and in the number of loaded cells that can modify the structure/porosity of collagen gels [35,36], salts (namely divalent anions and cations) may also influence macromolecular aggregates [37], since proteins fold into specific and functional three-dimensional structures as the result of specific interactions between amino acid chemical functional groups and the surrounding solvent.

We have investigated the effect of magnesium since there is an increasing interest in this ion as a component of biomaterials used for bone replacement [38] or as a possible therapeutic agent capable of counteracting ectopic calcification [22] or as component of mineral deposits in pathological calcification [24,25]. Interestingly, in the presence of MgCl_2_, we have demonstrated positivity to von Kossa staining. Moreover, in our experimental condition, Mg^2+^ was observed in mineral deposits together with other elements. The presence of Mg is consistent with earlier reports where magnesium was identified in the EDX spectra of calcified valves [39] and can be incorporated, possibly with sodium and carbonate, during formation of calcium phosphate crystals [40]. Although discrimination among different apatitic deposits was beyond the scope of this study, formation of minerals with different composition, as in #C and #D, can better mimic the *in vivo* condition [41].

These findings seem in contrast with recent *in vitro* data excluding a physicochemical role of Mg^2+^ in inhibiting crystal growth or in altering calcium phosphate crystal structure and composition in vascular smooth muscle cell cultures [42]. This discrepancy can be explained by the different mineralized context (3D collagen gel *vs*. 2D cellular monolayer) and the method used to generate mineral deposits (enzymatic induction *vs*. Pi supplementation). Moreover, it has been reported that Mg^2+^ salts can modify collagen assembly [23]. Consistently, we have noticed a striking effect of MgCl_2_ on size and organization of collagen fibrils. Formation of a different porous structure could impact fibroblast’s behavior and cell morphology [43]. Gel retraction, for instance, does not seem to be affected by the presence of mineral deposits *per se* within the 3D structure, whereas it is dependent on gel structure, being lower in the presence of a dense matrix, as in condition #C, and higher in the presence of large pores, as in condition #D, as already observed by other Authors [44].

Moreover, in #D, although cells were less elongated compared to other experimental conditions, collagen gel was more retracted. Previous studies suggested that in fibroblasts a round shape may result from the inability of the collagen matrix to resist the force of cell contraction [45,46]. Additionally, Tamariz *et al.* [47] suggested that adhesive interactions of dendritic extensions were an indication of local remodeling of the collagen matrix, while the stellate/bipolar morphology was a consequence of reorganization and simplification of cell extensions during global remodeling of the whole gel.

## 4. Experimental Section

### 4.1. Preparation of Collagen Gels

Collagen gels were prepared according to manufacturer’s instructions (Gibco, Invitrogen, Milan, Italy). Briefly, a solution of type I collagen from rat tails, at the concentration of 2 mg/mL, was mixed with DMEM 5X (Gibco) and 0.025 N NaOH and allowed to be polymerized at 37 °C for 30 min (Figure 1). This basal condition is referred to as condition #A. Moreover, during gel preparation, the following components were added: 2.5 mM β-glycerophosphate (β-GP, Sigma-Aldrich, St. Louis, MO, USA) and 1.25 mM CaCl_2_ (BDH AnalR, Milan, Italy) as potential sources of P and Ca ions necessary to form apatitic deposits (condition #B); β-GP, CaCl_2_ as in condition #B, but in the presence of 1 U/mL Alkaline Phosphatase from bovine intestinal mucosa (ALP, Sigma-Aldrich, St. Louis, MO, USA), since this enzyme is required to cleave P from substrates (condition #C). Finally, to the mixture, as in condition #C, different concentrations of MgCl_2_ (Riedel-DE Haen, Seelze, Germany) were added (condition #D). As a result of a set of preliminary experiments, a working solution of 62.5 mM MgCl_2_ was used.

In addition, 400 μL of each collagenous mix was quickly put in a 24-well-plate and, after polymerization, gels were gently detached before adding 500 μL of DMEM. Where required, human dermal fibroblasts, at the concentration of 1.2 × 10^5^ cells/mL, were added to collagen solutions before gel polymerization. In order to allow cells to settle in the 3D structure, all gels were allowed to stabilize for 24 h before morphological analyses.

### 4.2. Scanning (Low Vacuum Mode) and Scanning Transmission Electron Microscopy (SEM and SEM-STEM) and Energy-Dispersive Spectroscopy (EDS)

After dehydration and air drying, without any further treatment, samples were observed by environmental scanning electron microscopy (Nova NanoSEM 450, FEI, Hillsboro, OR, USA). Accelerating voltage was 5 kV for morphological observations and 20 kV for micro-analytical analyses. A gaseous analytical detector (GAD) for backscattered electrons and a large field detector (LFD) for secondary electrons were used for imaging. Microanalysis was performed using X-EDS (Quantax 200, Bruker, Berlin, Germany). Collagen fibril diameters were evaluated using the Measure and Label plugin for ImageJ. For each experimental condition, at least 30 collagen fibrils from two different areas of the gel (magnification = ×10,000) were measured in duplicate.

For STEM observations, collagen gels were routinely fixed in 2.5% glutaraldehyde in phosphate-buffered saline (PBS), post-fixed in 1% osmium tetroxide, dehydrated and embedded in Spurr resin. Ultrathin sections stained with uranyl acetate and lead citrate were observed by SEM-STEM (Nova NanoSEM 450-STEM).

### 4.3. Histochemistry and Light Microscopy

Collagen gels were fixed with 10% neutral buffered formalin, dehydrated and paraffin embedded. Sections, 4-μm thick, were obtained with a LEICA microtome and immobilized on glass slides (Wetzlar, Germany). After deparaffinization, samples were stained with Anilin blue and observed by light microscopy (Zeiss, Jena, Germany). Von Kossa staining was applied to detected mineralization [31,32]. Briefly, sections were placed in demineralized water and exposed to 5% silver nitrate solution for 30 min under UV irradiation. After rinsing in demineralized water, sections were exposed to 2% sodium thiosulfate for 5 min, rinsed again in demineralized water, dehydrated and covered with glass coverslips for microscopic analyses. Experiments were performed three times in duplicate.

### 4.4. Gel Retraction and Live/Dead Assay

Gels were prepared as described above. Human dermal fibroblasts (HDF), purchased from Thermo Fisher Scientific (Waltham, MA, USA), were routinely grown in DMEM supplemented with 10% foetal bovine serum (FBS) (Gibco-Thermo Fisher Scientific, Milan, Italy) according to standard procedures [48]. HDF were added to gels at a final seeding density of 1.2 × 10^5^ cells/mL in 2 mg/mL of collagen. In gel retraction experiments, cell-seeded collagen gels were prepared in duplicate in 35 mm diameter Petri dishes and evaluated at different time points (0, 6, 24 and 48 h). After 30 min at 37 °C, polymerized gels were immersed in 2 mL of standard culture medium (DMEM supplemented with 10% FBS) and detached from the wells using a small spatula. Addition of serum is a fundamental requirement, since, in its absence, cells are not able to retract [35,36]. Three independent experiments were carried out in duplicate and free-floating gel retraction was assessed at the indicated time points as a percentage of the initial gel area.

Cell viability was assessed by the “live/dead staining” using calcein-AM (Sigma, St. Louis, MO, USA) and propidium iodide (Sigma, St. Louis, MO, USA). Calcein-AM is a non-fluorescent, hydrophobic compound that easily permeates intact, live cells. The hydrolysis of calcein-AM by intracellular esterases produces calcein, a hydrophilic, strongly fluorescent compound that is well-retained in the cell cytoplasm.

Propidium iodide (PI) is impermeable to cells with an intact plasma membrane, hence when the cell integrity becomes compromised, it gains access to the nucleus, where it complexes with DNA, rendering the nucleus highly fluorescent [27].

Collagen gels were rinsed in PBS, incubated for 10 min at room temperature with 2 mL of calcein-AM (5 μM in PBS) and of propidium iodide (1 mg/mL in PBS). Cells were washed twice with PBS and observed by fluorescence microscopy at 24 and 48 h after seeding.

### 4.5. Statistical Analysis

Data were expressed as mean values ± SD/SEM of all measurements and compared by ANOVA test with significance at *p* < 0.05. Statistical data were obtained using GraphPad software, version 5.0 (San Diego, CA, USA).

## 5. Conclusions

In conclusion, this study demonstrates that, in a short time, an enzymatically mineralized collagen gel can be prepared in which mineral deposits and viable cells are homogeneously distributed. The effect of MgCl_2_ on collagen fibril assembly and organization highlights the importance of this ion in modifying matrix structural characteristics. The development of structurally different 3D mineralized collagen matrices, together with the use of fibroblasts, as prototype of sot connective tissue mesenchymal cells, may pave new ways for the study of ectopic calcification.

## Supplementary Materials

The following are available online at www.mdpi.com/1996-1944/9/6/477/s1.
