# Supplementary material for: Magnesium Modifies the Structural Features of Enzymatically Mineralized Collagen Gels Affecting the Retraction Capabilities of Human Dermal Fibroblasts Embedded within This 3D System
Source: Materials (Basel). 2016 Jun 15;9(6):477. doi: 10.3390/ma9060477 (PMC5456744; doi:10.3390/ma9060477)
Supplement: Supplementary file 1 [file materials-09-00477-s001.pdf]

Supplementary Materials: Magnesium Modifies the Structural Features of Enzymatically Mineralized Collagen Gels Affecting the Retraction Capabilities of Human Dermal Fibroblasts Embedded within This 3D System

Federica Boraldi, Angelica Bartolomeo, Giulia Annovi, Romain Debret and Daniela Quaglino

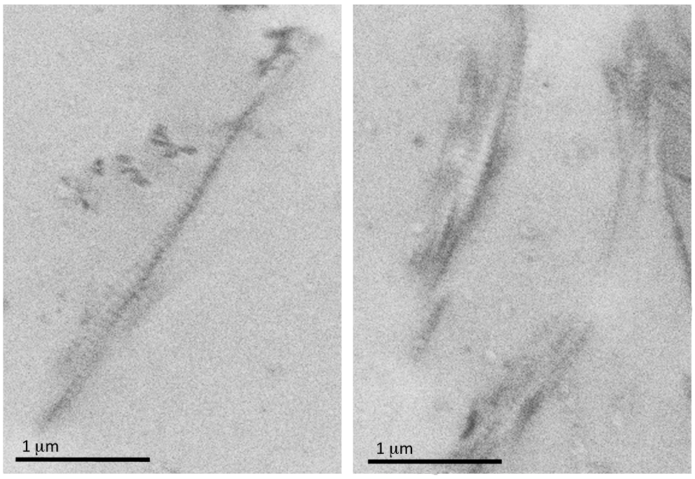

Figure S1. Scanning Transmission Electron Microscopy (SEM-STEM) of collagen fibrils in two different experimental conditions (condition #A, **left** and condition #D, **right**).

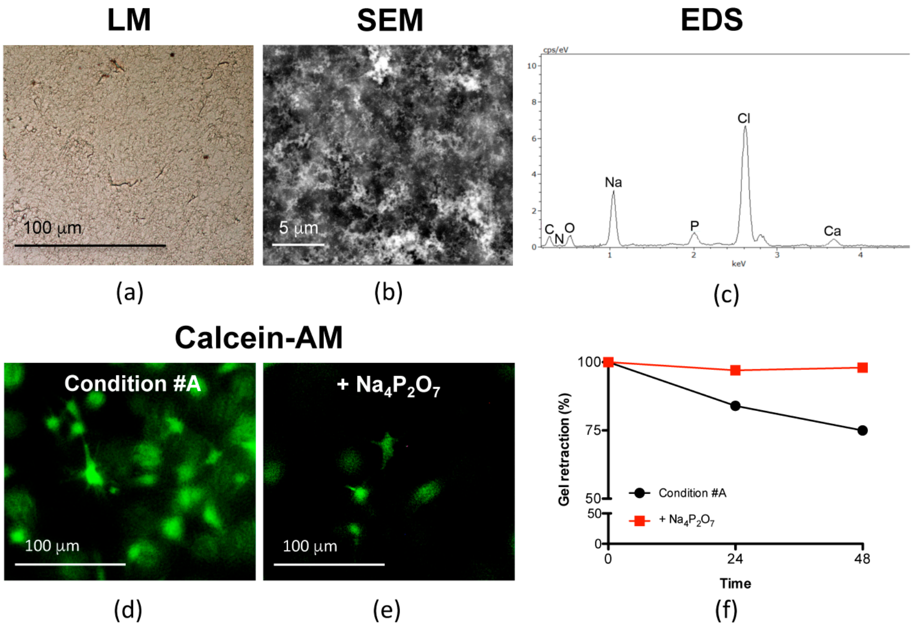

Figure S2. Mineralized collagen gel was prepared providing $CaCl_2$ and $Na_4P_2O_7$. (**a**) The presence of mineral deposits is visualized by Light Microscopy (LM) after von Kossa staining (brown); (**b**,**c**) Shape and composition of mineral deposits is shown by Scanning Electron Microscopy (SEM) and Energy-Dispersive Spectroscopy spectra (EDS), respectively; (**d**,**e**) Cell viability was assessed by calcein-AM staining; (**f**) C ollagen retraction was measured and data compared with those obtained in condition #A.
